# Safety Profiles Related to Dosing Errors of Rapid-Acting Insulin Analogs: A Comparative Analysis Using the EudraVigilance Database

**DOI:** 10.3390/biomedicines12102273

**Published:** 2024-10-07

**Authors:** Ioana Rada Popa Ilie, Andreea Loredana Vonica-Tincu, Carmen Maximiliana Dobrea, Anca Butuca, Adina Frum, Claudiu Morgovan, Felicia Gabriela Gligor, Steliana Ghibu

**Affiliations:** 1 Department of Endocrinology, Faculty of Medicine, “Iuliu Haţieganu” University of Medicine and Pharmacy, 3-5 Louis Pasteur Street, 400349 Cluj-Napoca, Romania; ioana.ilie@umfcluj.ro; 2 Preclinical Department, Faculty of Medicine, “Lucian Blaga” University of Sibiu, 550169 Sibiu, Romania; carmen.dobrea@ulbsibiu.ro (C.M.D.); anca.butuca@ulbsibiu.ro (A.B.); adina.frum@ulbsibiu.ro (A.F.); claudiu.morgovan@ulbsibiu.ro (C.M.); felicia.gligor@ulbsibiu.ro (F.G.G.); 3 Department of Pharmacology, Physiology and Pathophysiology, Faculty of Pharmacy, “Iuliu Haţieganu” University of Medicine and Pharmacy, 6A Louis Pasteur Street, 400349 Cluj-Napoca, Romania; steliana.ghibu@umfcluj.ro

**Keywords:** insulin analogs, rapid-acting insulin analogs, dosing errors, real-world evidence, pharmacovigilance

## Abstract

Insulin is essential for treating type 1 diabetes and insulin-requiring type 2 diabetes. Background/Objectives: Diabetes is a widespread condition that can lead to multiple and severe complications. Rapid-acting insulin analogs (RAIAs) and long-acting insulin analogs are prescribed for the effective management of diabetes. RAIAs are expected to be associated with a higher number of dosing errors because of their rapid onset, short duration of action, and the need for frequent dosing, compared to other insulin analogs. There are three approved RAIAs on the market: insulin lispro (LIS), insulin aspart (ASP), and insulin glulisine (GLU). The aim of this study is to evaluate the real-world evidence on dosing errors reported for RAIAs in EudraVigilance (EV), an established pharmacovigilance database, in comparison to other insulin analogs and human insulins. Methods: A descriptive analysis and a disproportionality analysis were conducted. Results: ASP and LIS were associated with high percentages of adverse drug reactions (ADRs) (22% and 17%, respectively), with over 70% of the reports involving serious ADRs. A higher frequency of cardiac and eye disorder ADRs was observed for LIS compared with ASP and GLU. GLU showed a higher frequency of ADRs in the skin and subcutaneous tissue disorders category. LIS dosing errors accounted for 5% of the total number of cases, while dosing errors for ASP and GLU were less than 3%. The most frequently reported dosing errors involved improper dosing (49%). Conclusions: Although there were fewer dosing errors of RAIAs in comparison to other insulins, the severity of the potential outcome highlights the importance of precise dosing and timing. Improved the monitoring and reporting of these dosing errors could enhance diabetes patient care. Additionally, smart medical devices could improve therapeutic outcomes.

## 1. Introduction

According to the International Diabetes Federation, more than 537 million people around the world have diabetes [1]. An alteration in glucose and lipid metabolism leads to metabolic syndrome and alters the individual’s health status [2,3]. Exogenous insulin is the essential treatment for patients with type 1 diabetes and for patients with insulin-requiring type 2 diabetes, underscoring the critical role of insulin and its analogs in managing the disease. Although it has been 100 years since the first insulin preparation was injected into humans and 28 years since the approval of insulin lispro, the first rapid-acting insulin analog (RAIA) [4], insulin replacement remains a challenging task both in the interprandial fasting state and at mealtime, when rapid-acting insulin must be provided [5,6]. Improving postprandial glucose (PPG) control with RAIAs is particularly challenging and often more difficult than optimizing glucose levels in the fasting state with basal insulin. Nowadays, RAIAs represent the ideal insulin preparations at mealtime. The American College of Obstetricians and Gynecologists and the Endocrine Society recommend insulin aspart and insulin lispro administration instead of regular insulin in diabetic patients during pregnancy [7].

Human insulin has a slower onset and a longer duration of action compared to the body’s natural insulin response [8]. RAIAs were developed to address the slow subcutaneous absorption of regular human insulin (RHI). Three RAIAs have emerged to date, namely insulin lispro (LIS), insulin aspart (ASP), and insulin glulisine (GLU). These agents effectively minimize postprandial glucose excursions and reduce both 2 h postprandial plasma glucose (PP-PG) levels and the risk of late hypoglycemia compared to RHI [5]. They are utilized as an additional treatment ahead of meals in combination with basal insulins, to mimic the body’s natural insulin response postprandially or to supplement oral hypoglycemic agents [9,10]. RAIAs are absorbed quickly, reach their peak concentration quicker, and have a shorter duration of action compared to RHI, effectively managing glucose excursions after meals [10]. In clinical studies with patients with type 1 diabetes, RAIAs have shown a minor beneficial impact on lowering glucose (reduced %HbA1c) but with a diminished incidence of hypoglycemia as compared to RHI [11,12]. Moreover, a meta-analysis including twenty-seven studies published between 1999 and 29 June 2016 and involving more than 7000 patients found that RAIAs are more effective than RHI at reducing postprandial glucose and improving HbA1c in patients with type 1 diabetes [13].

However, there are important challenges and complexities associated with dosing these medications.

Dosing errors can vary depending on the type of diabetes medication due to several factors, including the complexity of the dosing regimen, the delivery method, the availability of advanced dosing tools, and the patient’s familiarity with the medication. A complex therapy that requires intricate titration and monitoring, such as a regimen involving multiple daily insulin injections, can increase the risk of errors. The delivery method, whether through insulin injections, insulin pens, or oral medications, also plays a role. For instance, insulin requires precise dosing and frequent adjustments based on carbohydrate intake, physical activity, and blood sugar levels, which might lead to more frequent errors compared to fixed-dose oral medications [14,15,16].

It is becoming increasingly apparent that the incorrect administration of insulin potentially contributes to an increased risk of unexpected hypoglycemia, glucose variability, and failure to achieve the desired glycemic goal [16,17].

Such errors in insulin therapy can potentially arise at every step, starting from the clinician’s office during the initial prescription, through transcription and dispensing, up to the point of administration. The majority of these errors occur during the administration stage [14,18,19]. Moreover, human factors—whether in a hospital or at home—can significantly impact the accuracy and safety of medication administration, thereby contributing to dosing errors. For example, a recent study by Ashley-Fenn et al. (2024) revealed that the risk of hospital insulin prescription errors in the UK has remained unchanged despite the adoption of several national initiatives. The audit, conducted on the clinical notes of 85 patients with diabetes, showed that one in four patients on insulin is affected by a prescription error within 48 h of hospital admission. The risk of error was higher for patients admitted outside of regular hours, particularly overnight, and this risk was not mitigated by the seniority of the admitting clinician or the expertise of the first consultant review [20].

On the other hand, patients may lack the medical knowledge to accurately calculate doses or may misinterpret instructions. Factors such as literacy levels, language barriers, experience with self-management, changes in medication products or formulations, access to proper dosing tools (like glucose monitors or insulin pens), and a poor understanding of the medication regimen and pen usage can significantly affect dosing accuracy [15,18,21].

These issues can contribute to varying error rates across different medications and types of insulin.

RAIAs are more prone to dosing errors, particularly overdoses, because of their rapid onset, short duration of action, and the need for frequent dosing, compared to other insulin analogs. Hypoglycemia is the most common adverse effect of insulin analogs detected in about 30% diabetic patients [22]. Hypoglycemia reduces the quality of life of patients with diabetes and increases the mortality and morbidity rate [23]. RAIAs such as ASP and LIS have demonstrated an approximately 20% diminished risk of hypoglycemic episodes compared to RHI [24,25]. Additionally, LIS was associated with an important reduction in nocturnal hypoglycemia in comparison to RHI [26].

In addition to hypoglycemia, other common side effects associated with RAIAs often arise from inconsistencies in dosing frequency and the amount of insulin administered. These side effects include glucose variability or hyperglycemia, difficulty in walking, general fatigue, skin rashes, bilateral leg edema, blurred vision, QT interval prolongation, cardiac arrhythmias, and the ‘dead in bed’ syndrome [10,27]. The ‘dead in bed’ syndrome is linked to severe nocturnal hypoglycemia-induced cardiac dysrhythmias in diabetic patients [28]. Additionally, insulin-related weight gains are also reported as adverse effects. Moreover, insulin injection could cause discomfort locally at the site of injection, namely pain, skin infection, and abscesses [10].

Thus, treatment with analogs enhances and balances glycemic control, reduces the risk of hypoglycemia, and offers greater dosing flexibility, leading to increased patient convenience. Nevertheless, there remains room for progress in dosing frequency, nocturnal glycemic control, and rapid prandial action to more closely align them with natural human insulin physiology [10]. In these circumstances, as with any medication, the evaluation of the adverse drug reaction (ADR) profiles of RAIAs through real-life surveillance and monitoring dosing errors for RAIAs is essential for ensuring diabetic patient safety and optimizing treatment outcomes.

The aim of this study is to analyze the post-marketing pharmacovigilance data of RAIAs and compare their safety information with other insulin analogs and human insulins, providing insights into the prevalence and nature of dosing errors.

## 2. Materials and Methods

### 2.1. Study Designs

A pharmacovigilance study on dosing errors related to RAIAs (ASP, GLU, and LIS) was conducted using data from EudraVigilance (EV). Dosing errors were classified as overdosing, underdosing, and improper dosing. All Individual Case Safety Reports (ICSRs) submitted to EV up until 28 July 2024 were analyzed. The data were extracted from the electronic database available at https://www.adrreports.eu/ [29], between 29–31 July 2024.

### 2.2. Material

In the EV database, drug dosing errors are classified under the “injury, poisoning, and procedural complications” System Organ Class (SOC) according to the Medical Dictionary for Regulatory Activities (MedDRA). For this present study, 24 preferred terms (PTs) [30] were used to identify dosing errors related to insulins. Table 1 presents the PTs grouped into three categories of dosing errors: improper dosing, overdosing, and underdosing errors. Errors that do not specify in what way the dose was improperly modified were included in the improper dosing category. Additionally, all PTs referencing “dose omission” were categorized as underdosing errors. According to the EMA “Good practice guide on the recording, coding, reporting, and assessment of medication errors”, an overdose refers to administering more than the maximum recommended dose (in terms of quantity and/or concentration), whereas an underdose is the administration of less than the minimum recommended dose (in terms of quantity and/or concentration) [31]. The dosing errors category does not include PTs referring to intentional overdosing, intentional underdosing, or intentional improper dosing. The premixed combination of ASP and DEG was not included in the RAIAs category.

### 2.3. Descriptive Analysis

In the first part of the descriptive analysis, all reports submitted for RAIAs, longer-acting insulin analogs (degludec—DEG, detemir—DET, and glargine—GLA), fixed combinations of insulins (ASP-DEG, GLA-LIX), and RHI were considered. The distribution of ICSRs by insulin type and the characteristics of ICSRs reported in EV, such as age, sex, origin (European Economic Area—EEA and non-EEA), and reporter category (healthcare professionals (HPs) and non-HP) were analyzed. A comparison was made between the average number of ADRs reported per ICSR for each insulin group. Additionally, a comparison of the severity of cases reported in EV was performed for the entire insulin group. An adverse reaction is considered serious if it results in death, persistent or significant disability or incapacity, or birth defect, is life-threatening, or requires hospitalization or prolongation of existing hospitalization [32].

In the second part of the descriptive analysis, a comparison between the three RAIAs was conducted. Thus, the distribution of ADRs reported for each insulin and by SOCs were calculated. The proportion of dosing errors among the total ADRs reported for the three RAIAs was also estimated. Finally, the distribution of the type of dosing errors and their outcomes was evaluated.

### 2.4. Disproportionality Analysis

Similarities and differences in the reporting probability of ADRs related to dosing errors were evaluated using disproportionality analysis. Following EMA recommendations, the Reporting Odds Ratio (ROR) was calculated using comparators from common therapeutic areas [33,34]. Thus, the analysis compared RAIAs (ASP, GLU, and LIS) with longer-acting insulin analogs (DEG, DET, and GLA), premixed insulin combinations (ASP-DEG, GLA-LIX), human insulin, and the entire insulin group. The ROR and 95% confidence intervals (CI) [30] were calculated with MedCalc software. Their odds ratio calculator [34] is available at https://www.medcalc.org/calc/odds_ratio.php (Version 20.123), (accessed on 9 August 2024).

According to EMA guidelines, a signal is considered disproportionate if there are at least 5 cases for each preferred term and the lower limit of the 95% CI exceeds 1 [35].

### 2.5. Ethics

Data published in EV are anonymous, thus no ethical approval was necessary.

## 3. Results

### 3.1. Descriptive Analysis

#### 3.1.1. Analysis of the Entire Insulin Group

**Figure 1:**
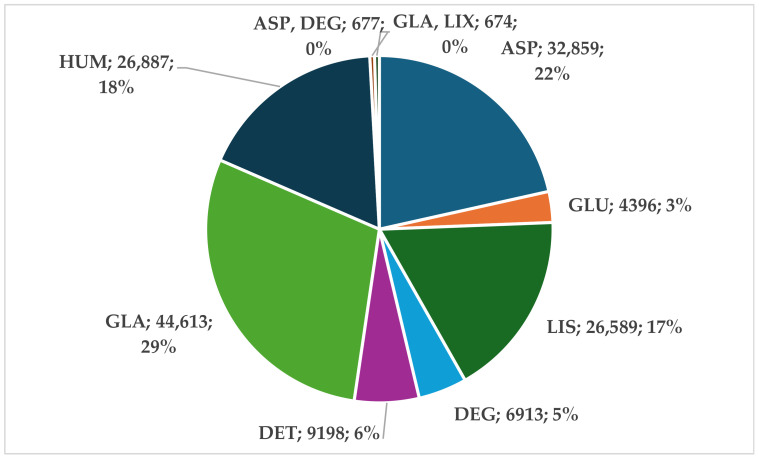
Distribution of ICSRs submitted in EV up to 28 July 2024. ASP—insulin aspartate; DEG—insulin degludec; DET—insulin detemir; GLA—insulin glargine; GLU—insulin glulisine; HUM—human insulin; LIS—insulin lispro; LIX—lixisenatide.

Up to 28 July 2024, a total of 152,806 ICSRs for insulins were uploaded in EV. For RAIAs, 63,844 ICSRs (41.8%) were reported, while 60,724 ICSRs (39.7%) were reported for long-acting analogs. RHIs accounted for 26,887 ICSRs (18%), and fixed combinations of insulin analogs were included in 1351 ICSRs (0.8%) (Figure 1).

According to data presented in Table 2, most ADRs cases related to insulin were reported in the 18–64 years group. Within this group, the highest proportion of ICSRs was for ASP (39.00%), GLU (36.80%), HUM (36.30%), and LIS (36.10%). In the 65–85 years group, the lowest proportion of ICSRs was reported for ASP (21.40%) and GLU (23.30%). An equal distribution of ICSRs for rapid analogs between the two sexes could be observed. Regarding the origin of patients, an equal distribution of cases between EEA and non-EEA regions could be observed only for ASP. Most cases reported in EV for LIS (29.30%), GLA (25.90%), HUM (27.00%), and premixed ASP insulin with DEG (9.70%) originated from non-EEA regions. Generally, the proportion of ICSRs reported by HPs is similar to that reported by non-HPs.

**Figure 2:**
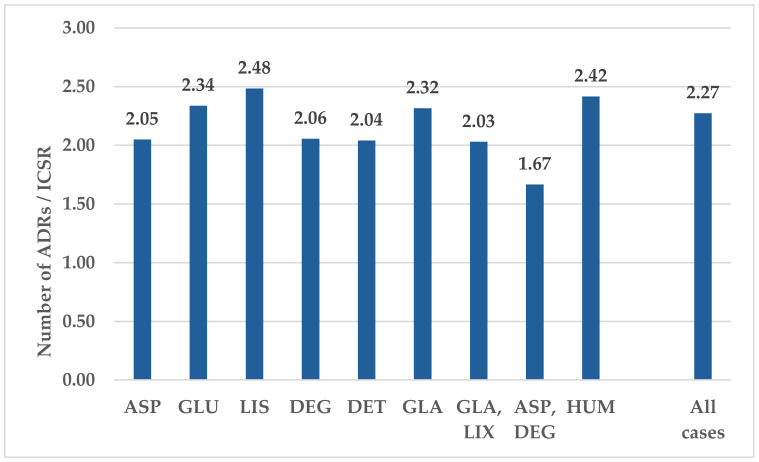
The average of ADRs reported in an ICSR. ASP—insulin aspartate; DEG—insulin degludec; DET—insulin detemir; GLA—insulin glargine; GLU—insulin glulisine; HUM—human insulin; LIS—insulin lispro; LIX—lixisenatide.

Figure 2 shows the average number of ADRs reported per ICSR. Overall, the average is 2.27 ADRs per ICSR. LIS shows the highest value in the series (2.48), closely followed by HUM (2.42). GLU also has a higher average (2.34) compared to the overall cases, whereas ASP has a lower average (2.05), similar to the long-acting analogs DEG (2.06) and DET (2.04).

**Figure 3:**
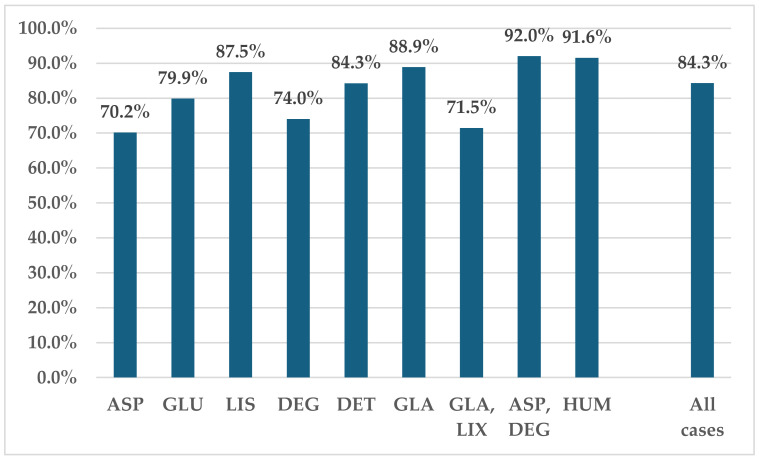
Serious ADRs reported for insulins in EV. ASP—insulin aspartate; DEG—insulin degludec; DET—insulin detemir; GLA—insulin glargine; GLU—insulin glulisine; HUM—human insulin; LIS—insulin lispro; LIX—lixisenatide.

Of the total ADRs, 84.3% were classified as serious. In the RAIAs group, GLU (79.9%) and ASP (70.2%) had lower percentages of serious ADRs, while LIS (87.5%) had a higher percentage, similar to GLA (88.9%) and HUM (91.6%) (Figure 3).

#### 3.1.2. Analysis of Rapid-Acting Insulin Analogs (RAIAs)

**Figure 4:**
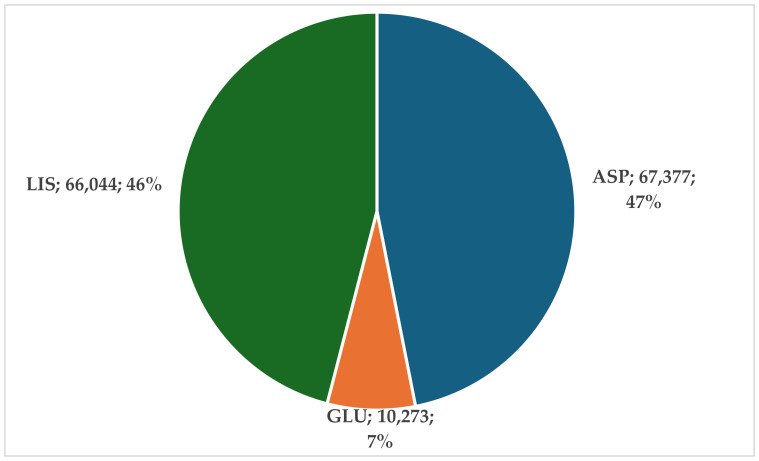
ADRs reported in EV for rapid analogs. ASP—insulin aspartate; GLU—insulin glulisine; LIS—insulin lispro.

For the three RAIAs, a total of 143,694 ADRs were registered in EV: 46.9% for ASP, 46.0% for LIS, and 7.1 for GLU (Figure 4)

Compared to ASP and GLU, for the LIS analog a higher frequency of ADRs in SOCs such as “cardiac disorders” (3.14%) and “eye disorders” (5.18%), but a lower frequency for “metabolism and nutrition disorders” (12.03%) could be noticed. Additionally, GLU had a higher frequency of ADRs in the “skin and subcutaneous tissue disorders” SOC (4.31%) (Table 3).

**Figure 5:**
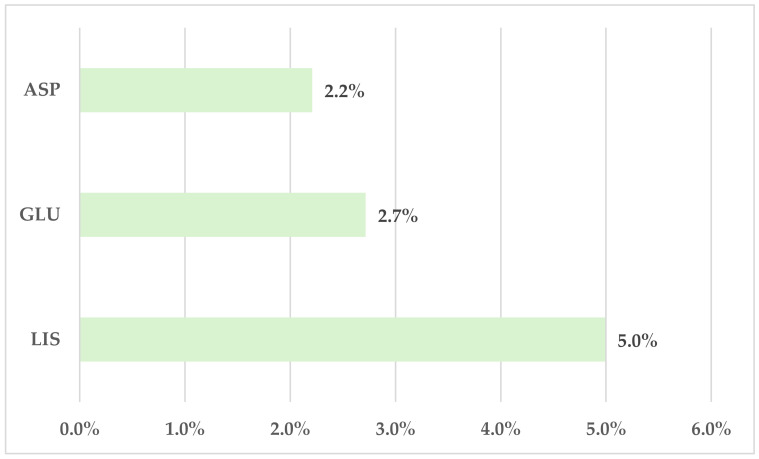
Frequency of dosing errors in total ADRs reported for rapid-acting insulin analogs. ASP—insulin aspartate; GLU—insulin glulisine; LIS—insulin lispro.

Dosing errors represented 5.0% of the total ADRs reported in EV for LIS. Lower frequencies were registered for ASP (2.2%) and GLU (2.7%) (Figure 5).

**Figure 6:**
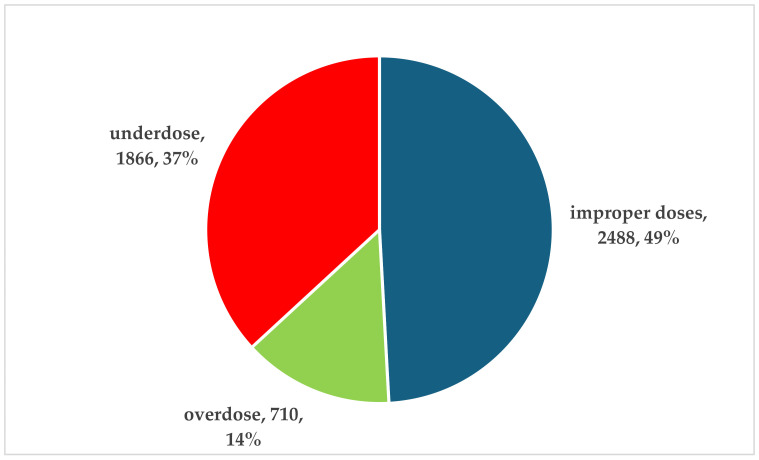
Distribution of dosing errors of rapid-acting insulin analogs by type of error.

Improper doses of RAIAs were reported in more than 49% of total dosing errors (*n* = 2488). Underdosing errors were reported in a high proportion (37%, *n* = 1866), while overdoses were reported in only 14% of cases (*n* = 710) (Figure 6).

**Figure 7:**
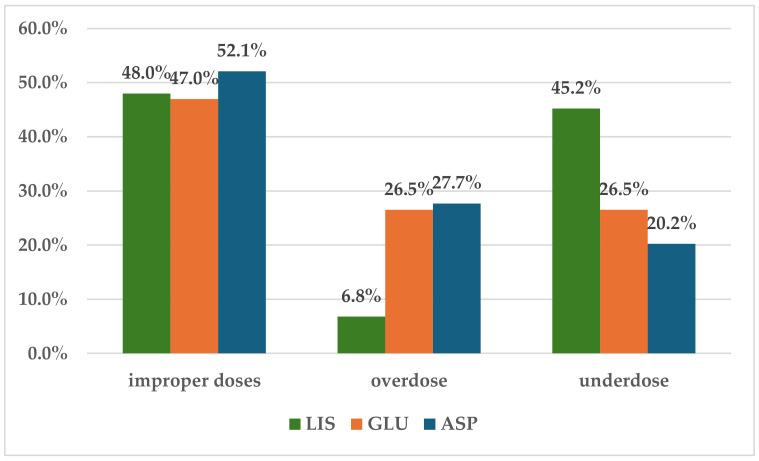
Distribution of dosing errors by category. ASP—insulin aspartate; GLU—insulin glulisine; LIS—insulin lispro.

According to data presented in Figure 7, the most frequent dosing errors were represented by improper doses related to RAIAs (47% for GLU, 48% for LIS, and 52.1% for ASP). LIS overdosing errors (6.8%) occurred less frequently than those for ASP (27.7%) and GLU (26.5%), but underdosing errors for LIS had a higher frequency within the total dosing errors (45.2%) compared to the other two RAIAs (GLU—26.5%; ASP—202%).

According to data from EV, some cases of overdosing had a fatal outcome: LIS (4.5%) and ASP (3.2%). Additionally, one fatal outcome was reported for the improper dosing of LIS (0.1%) and ASP (0.3%), as well as for the underdosing of ASP (0.7%). No fatal outcomes were associated with dosing errors involving GLU. For underdosing errors, the highest frequency of not recovered or unresolved cases was reported in underdosing errors, with GLU at 9.5%, ASP at 7.6%, and LIS at 4.9%, respectively (Table 4).

### 3.2. Disproportionality Analysis

**Figure 8:**
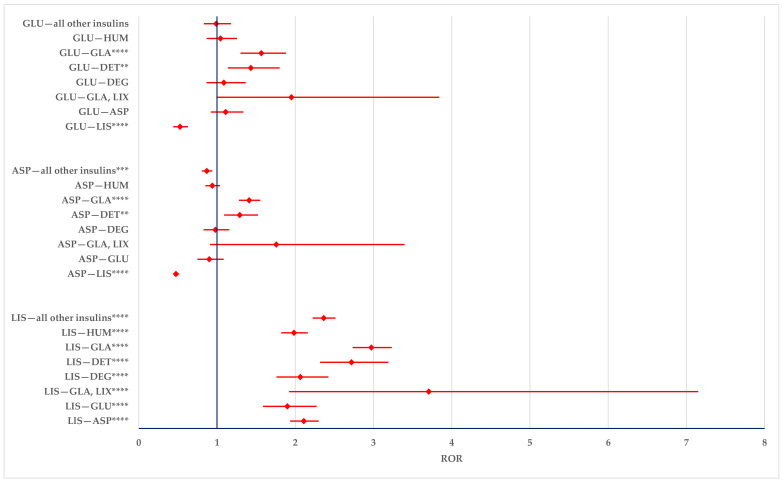
Modified doses. ASP—insulin aspartate; DEG—insulin degludec; DET—insulin detemir; GLA—insulin glargine; GLU—insulin glulisine; HUM—human insulin; LIS—insulin lispro; LIX—lixisenatide. ** *p* ≤ 0.01; *** *p* ≤ 0.001; **** *p* ≤ 0.0001.

According to the results presented in Figure 8, ADRs related to the improper dosing of LIS are more likely to be reported compared to the entire series and compared to all other drugs (ASP, GLU, DEG, DET, GLA, GLA-LIX, HUM). ASP and GLU also have a higher likelihood of being reported in comparison with DET (ASP: ROR—1.2893; 95% CI: 1.0904–1.5246 and GLU: ROR—1.4312, 95% CI: 1.1374–1.8008) and GLA (ASP: ROR—1.4099; 95% CI: 1.2784–1.5550 and GLU: ROR—1.5650; 95% CI: 1.3005–1.8834).

**Figure 9:**
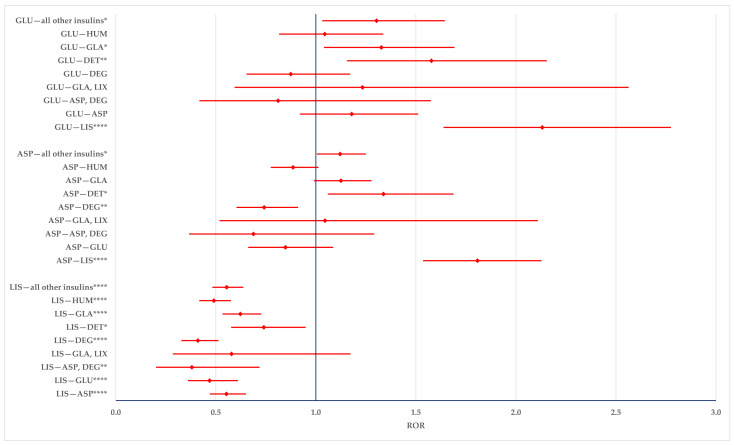
Overdosing errors. ASP—insulin aspartate; DEG—insulin degludec; DET—insulin detemir; GLA—insulin glargine; GLU—insulin glulisine; HUM—human insulin; LIS—insulin lispro; LIX—lixisenatide. * *p* < 0.05; ** *p* ≤ 0.01; **** *p* ≤ 0.0001.

The overdosing of LIS has a lower probability of being reported than all comparators except for GLA-LIX (ROR: 0.5785; 95% CI: 0.2852–1.1736). ASP and GLU have a higher probability of being reported compared to LIS (ASP: ROR—1.8078; 95% CI: 1.5358–2.1280 and GLU: ROR—2.1320; 95% CI: 1.6379–2.7751), DET (ASP: ROR—1.3376; 95% CI: 1.0596–1.6884 and GLU: ROR—1.5774; 95% CI: 1.1550–2.1544), and the entire group of all other insulins (ASP: ROR—1.1210; 95% CI: 1.0051–1.2503 and GLU: ROR—1.3033; 95% CI: 1.0322–1.6455). GLU is also more likely to be reported than GLA (ROR: 1.3271; 95% CI: 1.0405–1.6925) (Figure 9).

**Figure 10:**
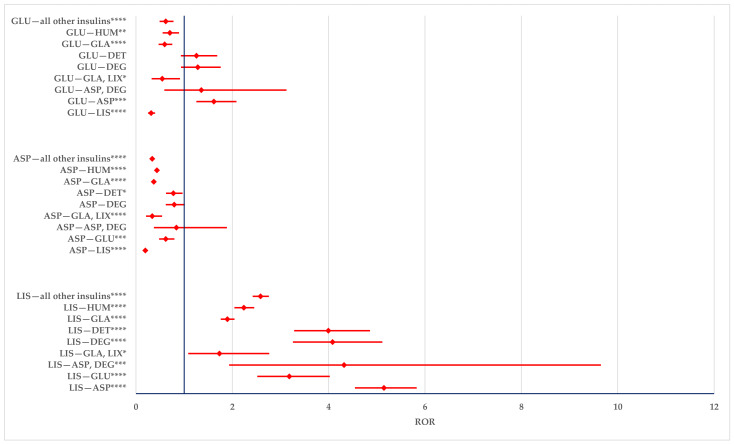
Underdosing errors. ASP—insulin aspartate; DEG—insulin degludec; DET—insulin detemir; GLA—insulin glargine; GLU—insulin glulisine; HUM—human insulin; LIS—insulin lispro; LIX—lixisenatide. * *p* < 0.05; ** *p* ≤ 0.01; *** *p* ≤ 0.001; **** *p* ≤ 0.0001.

Underdosing errors with LIS were reported with a higher probability compared to all other insulins. By contrast, underdosing errors with ASP were reported with a lower probability compared to all other insulins, except for DEG and ASP-DEG premixed insulin. GLU showed a higher probability of reporting in comparison to ASP (ROR: 1.6169; 95% CI: 1.2527–2.0869) and a lower probability of reporting compared to LIS (ROR: 0.3141; 95% CI: 0.2485–0.3971), GLA (ROR: 0.5960; 95% CI: 0.4710–0.7542), HUM (ROR: 0.7036; 95% CI: 0.5528–0.8954), and the group of all other insulins (ROR: 0.6185; 95% CI: 0.4910–0.7791) (Figure 10) could be observed.

## 4. Discussion

This study analyzed adverse reactions reported in EV for RAIAs. Most ICSRs (Figure 1) were reported for GLA, but ASP and LIS also had high percentages of RAs (22% and 17%, respectively). A previous study showed that GLA has a higher price and attractiveness compared to RAIAs, DET, or HUM [36]. Although efficient, and considered “standard care” [37], the difficulty in safely using first-generation basal insulin-like GLA 100 U/mL triggered the development of second-generation products (300 U/mL) that offer a higher stability of plasma concentrations and lower costs related to acute hospitalizations [37,38,39]. For ASP, the opinion of the scientific body is divided. Some reviews and meta-analyses report similar risks of hypoglycemia and adverse effects as for regular insulin [40], while others highlight the superiority of RAIAs [41]. The profiles of ASP and LIS are considered comparable in both efficacy and safety [42], supporting the evidence from EV.

Most frequently, ADRs were reported in the 18–64 and 65–85 age categories (Table 2). Insulin usage presents challenges to any age group. In the pediatric population, dosing errors often arise due to weight-based dosing calculations, which can be complex and prone to mistakes and the potential need for diluted insulin. Additionally, younger children may have difficulty communicating symptoms, leading to incorrect dosing adjustments [43].

Adults (18–64 years), particularly those with busy lifestyles, may experience dosing errors due to missed doses, incorrect timing, or difficulties in managing complex regimens. Stress and irregular schedules, as noted in emerging adults, can also contribute to these errors. Adolescents/emerging adults (18 to <25 years) were more likely to miss bolus doses compared with younger children and compared with older individuals (≥45 years) [44,45,46].

In older adults, factors such as cognitive decline, vision impairments, reduced mobility and dexterity, polypharmacy, reliance on others for meal timing, fear of hypoglycemia, and altered pharmacokinetics and pharmacodynamics elevate the risk of dosing errors and lead to varied responses to standard medication doses. While insulin treatment can cause adverse side effects like hypoglycemia and weight gain in any population, older adults with type 2 diabetes are particularly vulnerable to hypoglycemia [46,47].

Furthermore, biological differences between men and women, including body fat distribution (with women having more subcutaneous fat), hormonal variations, and metabolism, can influence how drugs are absorbed, distributed and metabolized, leading to different dosing needs and potential risks for dosing errors [21,48].

However, an equal distribution of ICSRs related to rapid analogs was observed between the two sexes.

Over 70% of the reports include serious ADRs (ASP 70%, GLU—80%, LIS—88%) (Figure 4). These figures underscore the importance of correct dosing and timing [49], as complex insulin regimens can be challenging for some patients [46]. For seniors over 60 years, severe insulin-related hypoglycemia and other ADRs often result in emergency consultations, hospitalizations, and an increased risk of mortality [50,51].

A recent cohort study in the USA found that more than nine cases of hypoglycemia per 1000 inhabitants per year resulted in emergency consultations and hospitalizations [52].

A higher frequency of cardiac and eye disorder ADRs was observed with LIS compared with ASP and GLU (Table 4, Supplementary Materials). However, a study on pregnant women found no association between LIS and the development of diabetic retinopathy [53]. The integrity of the inner blood–retinal barrier is important, thus its impairment contributes to the progression of diabetic retinopathy [54]. Insulin acts on the heart via the KB/Akt signal pathway [55], and ASP has shown cardiac benefits for diabetic patients [56]. This study found LIS had a lower frequency of metabolism and nutrition disorder ADRs compared to other RAIAs. The present study found LIS had a lower frequency of metabolism and nutrition disorder ADRs compared to other RAIAs. A meta-analysis reported similar metabolic control in both obese and non-obese diabetic patients treated with LIS [57]. Finally, LIS and ASP have been associated with a lower risk of hypoglycemic attacks [10].

GLU showed a higher frequency of ADRSs in the skin and subcutaneous tissue disorders category. An online survey of patients using RAIAs found that many experienced skin irritation and other adverse effects at the injection site during continuous therapy [58]. The frequent rotation of injection sites is recommended to avoid skin thickening [59].

From the pathophysiological point of view, an important aspect is the potential causality of ADRs by RAIAs. Based on the pharmacokinetic and pharmacodynamic profiles of the RAIAs, hypoglycemia is the most common and significant short-term ADR and is most likely directly caused by insulin. Other short-term ADRs for RAIAs include hyperglycemia, dizziness, sweating, or palpitations due to sudden changes in blood glucose levels, injection site reactions, allergic reactions, and edema. They can be directly attributed to the pharmacokinetic or pharmacodynamic effects of RAIAs. In summary, all three RAIAs—LIS, ASP, and GLU—exhibit similar profiles regarding the frequency and severity of hypoglycemia, injection site reactions, and allergic reactions, although minor differences exist, in particular in terms of the incidence of hypoglycemia, as already mentioned [27,42,60,61,62,63,64].

Additionally, patient-specific factors like dementia and cognitive impairment, renal and hepatic impairment, cardiovascular autonomic dysfunction, polypharmacy, diet and physical activity levels, and adherence to dosing schedules can all influence the likelihood of short-term ADRs. Therefore, it is crucial to distinguish between ADRs directly caused by the drug and those influenced by these predisposing factors [60,65,66,67,68,69,70].

Some ADRs, like neoplasms (cancers), may take years to manifest, making it difficult to establish a direct link between insulin use and the development of such conditions. Still, some studies suggest a positive association between insulin dose and the risk of malignant neoplasms in diabetes patients; this applies to both human insulin and insulin analogs [71].

However, other research indicates that although insulin analogs might promote tumor progression by upregulating mitogenic signaling pathways, there is no strong evidence that they, or human insulin, increase the risk of breast cancer [72].

When evaluating ADRs related to RAIAs, it is important to recognize that some ADRs may not be directly caused by insulin but rather may occur due to other factors or underlying conditions. ADRs that might be considered non-causal often include chronic skin disorders, autoimmune diseases, or cardiovascular events. Understanding these broader contexts and potential contributing factors is crucial for distinguishing between causal and non-causal relationships [73,74,75].

LIS dosing errors account for 5% of total cases, while ASP and GLU dosing errors each account for less than 3%. A literature review found that missed and mistimed doses are common in patients with both types of diabetes and could pose a clinically relevant problem [46]. The fear of hypoglycemia has been cited as a reason for delaying insulin boluses [76].

For the three RAIAs, the most frequently reported errors were improper dosing (49%) (without specifying the exact category) and underdosing (37%).

Overdosing errors accounted for only 14% of the total, with a higher frequency for ASP and GLU. Overdosing may sometimes be intentional, as demonstrated in multiple studies [77,78,79], including a case report of a patient with a history of depression and suicide attempts using both ASP and GLU [80]), and another case report involving a patient using GLA [81].

However, underdosing errors were significantly higher for LIS (45%) compared to GLU (27%) and ASP (20%). A retrospective observational study found that a high proportion of patients were non-adherent to treatment with RAIAs, possibly due to fear of hypoglycemic events, the complexity of the dosing schedule, or other factors relating to patient behavior [82].

Although death most frequently occurs with LIS (4.5%) and ASP (3.2%) overdoses, the majority of unresolved or unrecovered cases were associated with underdoses. A toxicological study showed that in two cases, self-poisoning with ASP led to hypoglycemia and caused death [83].

GLU underdoses (9.5%) were most frequently associated with unfavorable outcomes (unresolved/unrecovered) compared to LIS (4.9%) or ASP (7.6%) underdoses (Table 4). Hyperglycemia, a dangerous status resulting from insulin underdosing, can lead to multiple organ damage and complications [84,85].

The disproportionality analysis showed a higher probability of reporting improper doses and underdosing with LIS compared to all other insulins. However, overdoses of LIS had a lower probability of being reported compared to other insulins. GLU overdoses were reported with a higher probability than those for LIS, DET, GLA, or the entire group of insulins. For ASP, the probability of reporting overdoses was higher than for LIS, DET, or the entire group of insulins. ASP had a lower probability of reporting underdoses compared to the entire group of insulins, RHI, and each analog except for DEG. Conversely, GLU had a lower probability of reporting underdoses compared to RHI, LIS, GLA, and to the entire group of insulins. The effective treatment of diabetes is often complex, requiring different molecules and precise dosing [86,87]. A recent review and meta-analysis reported that the adherence to insulin treatment was lower than expected, at approximately 50% [84]. One promising solution for precision timing and accurate dosing would be the use of modern medical devices such as smart insulin pens [88,89].

### Strengths and Limitations of the Study

The study’s strengths lie in its impact on identifying and understanding real-world dosing errors related to the use of RAIAs as reported in the EV database. This is a crucial first step in developing strategies to reduce the number and/or severity of errors, thereby improving patient safety and disease management. DM is a complex condition, with significant implications for the healthcare system [90]. The optimal management of DM enhances patients’ quality of life and reduces the burden on healthcare resources.

Another advantage of this study is its systematic search in a comprehensive data source, recognized by international committees and authorities. EV is a reliable, open access data collection platform concerning the safety of medicines, operated by the European Medicines Agency, and is regularly updated [91].

A firm conclusion on the causality of adverse reactions cannot be drawn due to several limitations involving each stakeholder category: patients, reporting system, and medicinal products. One major limitation is the absence of an exact number of patients who received the drugs [92]. Common limitations recognized in pharmacovigilance databases stem from the raw data in individual reports, which often lack critical information such as patient age, comorbidities, and the concomitant use of other medications [93]. The spontaneous reporting methodology does not guarantee that all the ADRs are accounted for [30]. Additionally, variations in reporting are observed, often due to the tendency to find more ADR reports for newly approved drugs, medications with well-known active ingredients [92], and the influence of national and international policies on reporting [94,95]. The underreporting of ADRs is also a recognized issue [96]. As far as medicinal products are concerned, access to accurate data on the volumes of dispensed RAIAs for the studied period was limited.

## 5. Conclusions

Diabetes is a global epidemic that can lead to multiple and severe complications. Insulin is a critical treatment for patients with type 1 diabetes and those with type 2 diabetes who require insulin. Since its introduction over 100 years ago, insulin therapy has evolved with various modifications to the molecule and advancements in drug delivery devices. Both RAIAs and long-acting insulin analogs are prescribed for effective diabetes management. RAIAs, due to their rapid onset, short duration, and the need for frequent dosing, were anticipated to be associated with a higher number of dosing errors, particularly overdoses, compared to other insulin analogs. An evaluation of real-world data from the EV database on dosing errors related to RAIAs revealed a surprising contrast: despite the low incidence of dosing errors (5% or fewer cases) for the three approved RAIAs, over 70% of reports included serious ADRs. Among the RAIAs, LIS was associated with a higher frequency of cardiac and eye disorder ADRs, while GLU had a higher frequency of ADRs in the skin and subcutaneous tissue disorders category. Strict control over treatment parameters, including the type of insulin, posology, and administration timing, is essential for both efficacy and safety. The enhanced monitoring and reporting of insulin dosing errors could improve the health outcomes of diabetic patients. Additionally, modern medical devices have the potential to further improve therapeutic success by providing better monitoring and control of insulin doses.

## Figures and Tables

**Table 1:** Classification of preferred terms by dosing error categories. ADR—adverse drug reaction; PT—preferred term [30].

ADR	PT
Improper dose	Dose calculation error
	Dose calculation error associated with device
	Drug dose titration not performed
	Drug titration error
	Incorrect dosage administered
	Incorrect dose administered
	Incorrect dose administered by device
	Incorrect dose administered by product
	Incorrect product dosage form administered
	Product dosage form confusion
	Wrong dosage formulation
	Wrong dose
Overdose	Accidental overdose
	Extra dose administered
	Overdose
	Prescribed overdose
Underdose	Accidental underdose
	Drug dose omission by device
	Incomplete dose administered
	Prescribed underdose
	Product dose omission
	Product dose omission in error
	Product dose omission issue
	Underdose

**Table 2:** Characteristics of ICSRs reported for insulin products. ASP—insulin aspartate; DEG—insulin degludec; DET—insulin detemir; GLA—insulin glargine; GLU—insulin glulisine; HUM—human insulin; LIS—insulin lispro; LIX—lixisenatide.

	ASP	GLU	LIS	DEG	DET	GLA	HUM	ASP, DEG	GLA, LIX
	n	n	n	n	n	n	n	n	n
	%	%	%	%	%	%	%	%	%
Total cases	32,860	4396	26,589	6913	9198	44,613	26,887	677	674
	(100.00%)	(100.00%)	(100.00%)	(100.00%)	(100.00%)	(100.00%)	(100.00%)	(100.00%)	(100.00%)
Age category									
Not Specified	9511	1374	8228	2339	3131	14,408	7897	157	332
	(28.90%)	(31.30%)	(30.90%)	(33.80%)	(34.00%)	(32.30%)	(29.40%)	(23.20%)	(49.30%)
0–1 Month	71	13	95	8	42	74	140	0	0
	(0.20%)	(0.30%)	(0.40%)	(0.10%)	(0.50%)	(0.20%)	(0.50%)	(0.00%)	(0.00%)
2 Months–2 Years	100	6	59	17	16	51	92	0	0
	(0.30%)	(0.10%)	(0.20%)	(0.20%)	(0.20%)	(0.10%)	(0.30%)	(0.00%)	(0.00%)
3–11 Years	1255	104	421	118	226	529	443	3	0
	(3.80%)	(2.40%)	(1.60%)	(1.70%)	(2.50%)	(1.20%)	(1.60%)	(0.40%)	(0.00%)
12–17 Years	1439	119	433	149	205	608	463	11	0
	(4.40%)	(2.70%)	(1.60%)	(2.20%)	(2.20%)	(1.40%)	(1.70%)	(1.60%)	(0.00%)
18–64 Years	12,801	1618	9601	2349	3153	14,097	9763	215	164
	(39.00%)	(36.80%)	(36.10%)	(34.00%)	(34.30%)	(31.60%)	(36.30%)	(31.80%)	(24.30%)
65–85 Years	7026	1023	7011	1735	2194	13,312	7230	251	175
	(21.40%)	(23.30%)	(26.40%)	(25.10%)	(23.90%)	(29.80%)	(26.90%)	(37.10%)	(26.00%)
More than 85 Years	656	139	741	198	231	1534	859	40	3
	(2.00%)	(3.20%)	(2.80%)	(2.90%)	(2.50%)	(3.40%)	(3.20%)	(5.90%)	(0.40%)
Sex									
Female	16,449	2243	13,764	3724	5115	23,319	13,718	334	344
	(50.10%)	(51.00%)	(51.80%)	(53.90%)	(55.60%)	(52.30%)	(51.00%)	(49.30%)	(51.00%)
Male	14,501	1963	12,033	3025	3419	19,519	11,781	328	281
	(44.10%)	(44.70%)	(45.30%)	(43.80%)	(37.20%)	(43.80%)	(43.80%)	(48.40%)	(41.70%)
NS	1909	190	792	164	664	1775	1388	15	49
	(5.80%)	(4.30%)	(3.00%)	(2.40%)	(7.20%)	(4.00%)	(5.20%)	(2.20%)	(7.30%)
Geographic origin									
EEA	16,410	1822	7796	2845	3256	11,534	7251	66	259
	(49.90%)	(41.40%)	(29.30%)	(41.20%)	(35.40%)	(25.90%)	(27.00%)	(9.70%)	(38.40%)
Non-EEA	16,449	2574	18,793	4068	5942	33,079	19,636	611	415
	(50.10%)	(58.60%)	(70.70%)	(58.80%)	(64.60%)	(74.10%)	(73.00%)	(90.30%)	(61.60%)
Reporter									
HP	16,174	2429	12,073	3880	4664	18,712	15,324	444	488
	(49.20%)	(55.30%)	(45.40%)	(56.10%)	(50.70%)	(41.90%)	(57.00%)	(65.60%)	(72.40%)
Non HP	16,666	1956	14,494	3033	4532	25,670	11,409	233	186
	(50.70%)	(44.50%)	(54.50%)	(43.90%)	(49.30%)	(57.50%)	(42.40%)	(34.40%)	(27.60%)
NS	19	11	22	0	2	231	154	0	0
	(0.10%)	(0.30%)	(0.10%)	(0.00%)	(0.00%)	(0.50%)	(0.60%)	(0.00%)	(0.00%)

**Table 3:** Distribution of ADRs by SOC for rapid-acting insulin analogs. ASP—insulin aspartate; GLU—insulin glulisine; LIS—insulin lispro.

	ASP	GLU	LIS
Blood and lymphatic system disorders	0.24%	0.18%	0.37%
Cardiac disorders	2.00%	1.71%	3.14%
Congenital, familial, and genetic disorders	0.17%	0.12%	0.22%
Ear and labyrinth disorders	0.25%	0.33%	0.64%
Endocrine disorders	0.12%	0.18%	0.27%
Eye disorders	2.03%	2.67%	5.18%
Gastrointestinal disorders	2.51%	2.83%	3.19%
General disorders and administration site conditions	10.92%	12.56%	12.04%
Hepatobiliary disorders	0.44%	0.42%	0.59%
Immune system disorders	1.18%	1.28%	1.18%
Infections and infestations	2.39%	2.13%	3.47%
Injury, poisoning, and procedural complications	8.95%	11.85%	11.74%
Investigations	19.08%	13.66%	18.25%
Metabolism and nutrition disorders	18.66%	20.55%	12.03%
Musculoskeletal and connective tissue disorders	1.27%	1.86%	2.72%
Neoplasms benign, malignant, and unspecified (incl cysts and polyps)	0.85%	0.76%	1.88%
Nervous system disorders	7.61%	8.20%	9.69%
Pregnancy, puerperium, and perinatal conditions	0.98%	0.61%	0.65%
Product issues	10.40%	4.59%	0.32%
Psychiatric disorders	1.54%	2.73%	2.67%
Renal and urinary disorders	1.15%	1.34%	2.20%
Reproductive system and breast disorders	0.14%	0.23%	0.23%
Respiratory, thoracic, and mediastinal disorders	1.38%	1.69%	2.17%
Skin and subcutaneous tissue disorders	2.88%	4.31%	2.47%
Social circumstances	0.33%	0.44%	0.10%
Surgical and medical procedures	1.54%	1.46%	0.81%
Vascular disorders	0.97%	1.30%	1.76%

**Table 4:** Distribution of dosing errors with unfavorable outcomes. ASP—insulin aspartate; GLU—insulin glulisine; LIS—insulin lispro.

	Fatal Outcome			Not Recovered/Not Resolved		
	LIS	GLU	ASP	LIS	GLU	ASP
improper doses	0.1%	0.0%	0.3%	2.7%	2.3%	1.8%
overdose	4.5%	0.0%	3.2%	3.6%		2.2%
underdose	0.0%	0.0%	0.7%	4.9%	9.5%	7.6%

## Data Availability

Data are contained within the article.

## Supplementary Materials

The following are available online at https://www.mdpi.com/article/10.3390/biomedicines12102273/s1, Table S1: Distribution of ADRs by SOC for other types of insulin.
