# Supplementary material for: Safety Profiles Related to Dosing Errors of Rapid-Acting Insulin Analogs: A Comparative Analysis Using the EudraVigilance Database
Source: Biomedicines. 2024 Oct 7;12(10):2273. doi: 10.3390/biomedicines12102273 (PMC11504911; doi:10.3390/biomedicines12102273)
Supplement: Supplementary file 1 [file biomedicines-12-02273-s001.zip › biomedicines-3187007-supplementary.pdf]

Table S1. Distribution of ADRs by SOC for other types of insulin. DEG — insulin degludec; DET — insuluin detemir; GLA — inslulin glargine; HUM — human insulin; LIS — insulin lispro; LIX — lixisenatide.

	DEG	DET	GLA	GLA, LIX	ASP, DEG	HUM
Blood and lymphatic system disorders	0.40%	0.37%	0.33%	0.15%	0.53%	0.57%
Cardiac disorders	2.90%	2.68%	2.97%	2.12%	3.72%	3.28%
Congenital, familial and genetic disorders	0.19%	0.40%	0.13%	0.00%	0.09%	0.30%
Ear and labyrinth disorders	0.51%	0.45%	0.63%	0.58%	0.00%	0.44%
Endocrine disorders	0.20%	0.07%	0.21%	0.15%	0.09%	0.29%
Eye disorders	2.62%	2.93%	5.25%	2.63%	1.51%	4.21%
Gastrointestinal disorders	4.38%	3.13%	3.12%	12.94%	3.81%	3.30%
General disorders and administration site conditions	10.27%	12.63%	11.01%	10.96%	9.57%	10.59%
Hepatobiliary disorders	0.65%	0.65%	0.54%	0.66%	1.15%	0.84%
Immune system disorders	0.97%	1.89%	0.83%	1.61%	1.24%	1.74%
Infections and infestations	3.32%	3.34%	3.09%	2.63%	2.30%	3.33%
Injury, poisoning and procedural complications	10.27%	10.74%	11.27%	10.16%	7.98%	9.99%
Investigations	14.98%	14.82%	12.63%	13.08%	10.82%	14.36%
Metabolism and nutrition disorders	17.27%	12.31%	15.04%	13.74%	26.42%	15.04%
Musculoskeletal and connective tissue disorders	2.13%	2.07%	2.61%	1.75%	1.24%	2.42%
Neoplasms benign, malignant and unspecified (incl cysts and polyps)	1.57%	1.52%	1.78%	1.75%	1.60%	1.28%
Nervous system disorders	8.20%	8.80%	9.97%	8.33%	11.52%	10.29%
Pregnancy, puerperium and perinatal conditions	0.78%	2.40%	0.33%	0.07%	0.18%	1.36%
Product issues	4.16%	3.85%	2.78%	2.85%	3.10%	1.87%
Psychiatric disorders	2.62%	1.65%	2.62%	1.46%	1.42%	2.72%
Renal and urinary disorders	1.62%	1.75%	1.99%	2.41%	1.95%	2.05%
Reproductive system and breast disorders	0.27%	0.19%	0.23%	0.37%	0.09%	0.25%
Respiratory, thoracic and mediastinal disorders	2.05%	2.13%	2.06%	2.34%	1.24%	2.39%
Skin and subcutaneous tissue disorders	3.39%	4.53%	3.48%	4.17%	3.99%	3.54%
Social circumstances	0.65%	0.51%	0.81%	0.73%	0.27%	0.15%
Surgical and medical procedures	2.47%	2.73%	2.75%	1.54%	2.57%	1.22%
Vascular disorders	1.15%	1.46%	1.55%	0.80%	1.60%	2.15%
